# The safety of multiple oral immunotherapy in phase one studies at a single center

**DOI:** 10.1186/2045-7022-3-S3-O25

**Published:** 2013-07-25

**Authors:** TLR Dominguez, A Mehrotra, S Wilson, L Winterroth, A Blakemore, K Sampson, K Nadeau

**Affiliations:** 1 PEDS Allergy & Immunology, Stanford University, Stanford, CA, USA; 2 Stanford University, Stanford, CA, USA

## Background

There are over 15 million people with food allergies, 38% of which suffer from more than 1 food allergy. With increasing interest in oral immunotherapy (OIT) as a potential treatment, the safety of ingesting known allergens is a major concern. Little to no data are available for OIT with multiple simultaneous allergens. Also, the effect of omalizumab as adjunctive therapy in rush multi OIT had not been investigated.

## Methods

After obtaining IRB approval, subjects (pediatric and adults) were consented and enrolled in an ongoing multiple allergen OIT clinical studies: one phase 1 study “standard multi OIT” (n=27), and a second noted as “rush multi OIT” in which food allergen doses were 100x higher than “standard multi OIT” due to adjunct therapy with omalizumab (n=11). Food allergens included were milk, egg, peanut, walnut, cashew, almond, pecan, hazelnut, wheat, soy, and/or sesame. Up to 5 foods were allowed to be given in the OIT regimen simultaneously depending on the subjects’ allergic reactions to screening food challenges. Entry criteria for both studies were the same; they included a positive DBPCFC for each food allergen with positive skin prick test and/or positive food specific IgE >7kU/L for each food allergen. Subjects receiving omalizumab had injections between weeks 1 and 15. Subjects taking omalizumab and OIT were increased on their initial dose by up to 100 fold more than the initial dose given to subjects taking OIT without omalizumab. Safety was the primary outcome. The secondary goal of both studies was to reach 4000mg of each allergen.

## Results

These studies are still ongoing. At an average of 28 weeks into studies, there have been an estimated 7471 doses in the standard multiple OIT group, with 371 documented clinical reactions (4.9%). There were 150 documented clinical reactions occurring in an estimated 1076 doses (13.9%) in the rush multiple OIT group. Reactions were mostly mild in both groups (508 mild reactions out of 520 total reactions) and mainly consisted of abdominal pain, pruritus, and rash. No subjects withdrew due to adverse side effects. Six reactions (out of 8547 doses) required epinephrine, two in the rush multi OIT study and four in the standard multi OIT study.

## Conclusion

Multiple OIT should be performed in a carefully monitored hospital environment with well trained staff. More studies are needed to obtain data on short term and long term efficacy and safety of multi OIT to address the high unmet need of multisensitized food allergy patients.

## Disclosure of interest

None declared.

